# Translation termination-dependent deadenylation of MYC mRNA in human cells

**DOI:** 10.18632/oncotarget.25459

**Published:** 2018-05-25

**Authors:** Béatrice Jolles, Affaf Aliouat, Vérène Stierlé, Samia Salhi, Olivier Jean-Jean

**Affiliations:** ^1^ Sorbonne Université, CNRS-UMR8256, Biological Adaptation and Ageing, Institut de Biologie Paris Seine (B2A-IBPS), F-75252 Paris, France

**Keywords:** MYC, mRNA deadenylation, poly(A) tail, translation termination, eRF3

## Abstract

The earliest step in the mRNA degradation process is deadenylation, a progressive shortening of the mRNA poly(A) tail by deadenylases. The question of when deadenylation takes place remains open. MYC mRNA is one of the rare examples for which it was proposed a shortening of the poly(A) tail during ongoing translation. In this study, we analyzed the poly(A) tail length distribution of various mRNAs, including MYC mRNA. The mRNAs were isolated from the polysomal fractions of polysome profiling experiments and analyzed using ligase-mediated poly(A) test analysis. We show that, for all the mRNAs tested with the only exception of MYC, the poly(A) tail length distribution does not change in accordance with the number of ribosomes carried by the mRNA. Conversely, for MYC mRNA, we observed a poly(A) tail length decrease in the fractions containing the largest polysomes. Because the fractions with the highest number of ribosomes are also those for which translation termination is more frequent, we analyzed the poly(A) tail length distribution in polysomal fractions of cells depleted in translation termination factor eRF3. Our results show that the shortening of MYC mRNA poly(A) tail is alleviated by the silencing of translation termination factor eRF3. These findings suggest that MYC mRNA is co-translationally deadenylated and that the deadenylation process requires translation termination to proceed.

## INTRODUCTION

Cytoplasmic mRNA degradation is one of the post-transcriptional processes that modulate gene expression. Once exported to the cytoplasm, the mammalian messengers are either translated or degraded or stored. These processes are tightly intertwined and our knowledge of the sequence of events that determine the post-transcriptional fate of mRNA is largely incomplete. The early view of mRNA degradation process was that mRNAs are protected from degradation throughout the translation process and that disassembly of translating polysomes precedes mRNA decay, mRNA moving from polysomes into RNA granules, such as P-bodies, for degradation [1]. This simplistic model was recently challenged by the observations that, in normal growth conditions, decapped intermediates of mRNA degradation are associated with translating ribosomes in Drosophila and yeast cells [2, 3]. Additional experimental evidences seem to strongly argue that translational repression is not a prerequisite for mRNA decay. Using a reporter transcript, Funakoshi *et al.* [4] have shown a competitive interaction between the translation termination complex and the deadenylase complexes for binding to mRNA-bound Poly(A) Binding Protein (PABP). These authors suggest that, as translation termination proceeds, a translation termination complex is released from poly(A) tail-bound PABP and recruited to the translating ribosome. This allows a deadenylase complex to bind to the PABP molecule, now available for a new interaction, and thus to initiate poly(A) tail degradation [4]. Deadenylation is the initial and rate-limiting step for major mRNA degradation pathways [5]. Two cytoplasmic deadenylation complexes degrade the poly(A) tail: first the PAN2–PAN3 complex shortens the 3′ poly(A) tail to approximately 100 nt that are then trimmed by the TOB-CAF1-CCR4-NOT complex [6]. This coupling model leads to the speculative view that the poly(A) tail shortening is incremented at each translation termination event. According to this model, one PABP molecule is potentially eliminated at each termination event and therefore uncovers ~20 adenosine residues susceptible to deadenylase attack [4, 7]. However, as noted by Brook and Gray [8], most mRNAs are likely translated by many more ribosomes than required to evict all the poly(A) tail-bounded PABP molecules, suggesting that PABP removal does not occur at every termination event. This remark is particularly judicious for mRNAs with short poly(A) tail and/or long half-life. Alternative mechanisms of deadenylation involve the targeted recruitment of deadenylation complexes either by sequence-specific RNA-binding proteins (RBPs) or by the miRNA pathway [9]. In human cells, an example of regulated recruitment of the CAF1-CCR4-NOT complex is provided by the AU-rich element-mediated mRNA decay. The AU-rich elements (AREs) are the most common destabilizing elements found in the 3′ untranslated region (3′UTR) of short-lived mRNAs, such as the proto-oncogene MYC, a pleiotropic transcription factor and one of the most commonly overexpressed genes in human cancers [10]. Pioneer works have revealed that deadenylation and rapid cytoplasmic turnover of MYC mRNA was activated by AU-rich sequences present within its 3′UTR [11, 12]. It has been recently shown that mammalian MYC mRNA is degraded through a specific decay process involving an interaction between the TOB-CAF1 deadenylation complex and the sequence-specific RNA-binding proteins CPEB [13].

To date, only few examples of a direct correlation between translation efficiency and deadenylation have been identified. For instance, it has been shown that during the S and M phases of synchronized human cells, poly(A) tail length dynamics influences translational control for a subset of genes with cell-cycle functions [14]. In addition, it has long been known that deadenylation is a major means to silence translation in oocytes and early embryos [15, 16]. This was recently confirmed by the use of high-throughput methods for poly(A) tail length profiling such as TAIL-seq and PALseq [17, 18]. Using the PALseq method, Subtelny *et al.* [18] have shown that poly(A) tail length strongly correlates with translational efficiency in early stages of zebrafish and frog embryos. However, this coupling disappears in gastrulating embryos and is absent in the non-embryonic samples studied, including mouse liver and a sampling of mammalian cell lines. This latter result was confirmed by the global poly(A) tail distribution analysis performed by Chang *et al.* [17] showing that, in HeLa and NIH 3T3 cells, the poly(A) tail length is not correlated to translational rates, hence suggesting that deadenylation and translation are not coupled. MYC mRNA is one of the rare examples for which a shortening of the poly(A) tail during ongoing translation was proposed on the basis of [^35^S]methionine incorporation experiments performed with HeLa cells treated with protein synthesis inhibitors [19].

Using polysome profiling and poly(A) tail length analysis of mRNAs from the polysomal fractions, we show here that MYC mRNA is deadenylated concomitantly to its translation in the fractions containing the largest polyribosomes.

In contrast to MYC mRNA, but in agreement with the global analyses described above [17, 18], the poly(A) tail length of a set of control mRNAs was largely unaffected by the ribosome density. Furthermore, MYC translation-dependent deadenylation is alleviated by the silencing of translation termination factor eRF3, suggesting that co-translational deadenylation takes place only on the translated MYC mRNAs that have experienced termination.

## RESULTS

### Co-translational deadenylation of MYC mRNA

**Figure 1:**
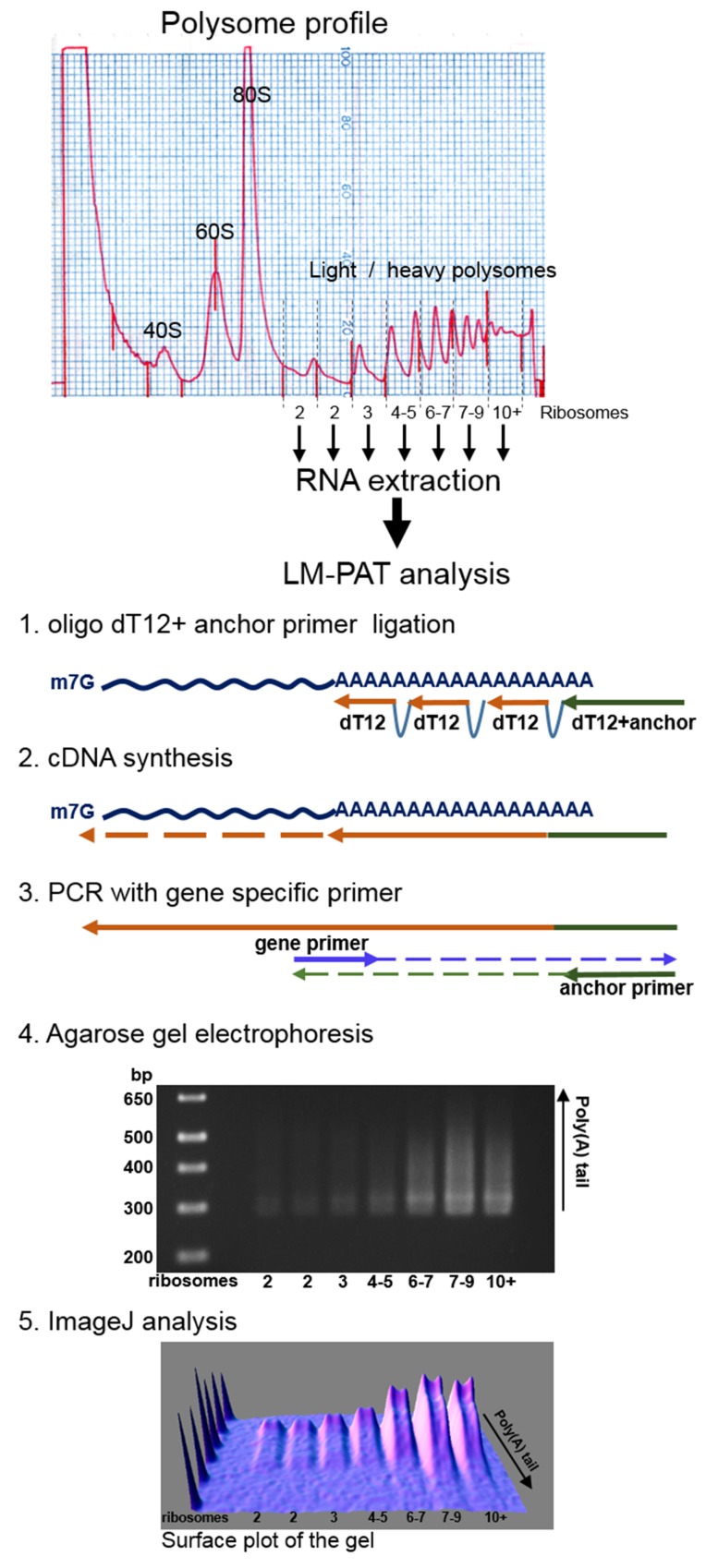
Schematic description of the experimental procedure Polysomes from human HCT116 cell extracts were separated by ultracentrifugation on a 15–50% sucrose gradient. RNA from polysomal fractions were isolated and subjected to Ligase-Mediated Poly(A) Test (LM-PAT). After amplification with gene specific forward primers of the resulting cDNA, the distribution of poly(A) tail lengths was visualized by agarose gel electrophoresis. The size of the DNA ladder fragments is indicated in base pairs on the left, the number of ribosomes in each fraction is indicated below the gel. 3D surface plot analysis of the agarose gel image was obtained using ImageJ software with minimum set to 0%, maximum to 100% and z-scale to 1.0.

In order to precisely evaluate the relationship between translation and deadenylation during normal cell growth, we have studied the poly(A) tail length of mRNAs engaged in an active translation process. The ribosomes of HCT116 human cells in the exponential growing phase were immobilized on mRNAs with cycloheximide, and cell extracts were loaded onto 15–50% sucrose gradients and subjected to centrifugation to obtain the polysome profiles (Figure 1). RNAs were then extracted from the polysomal fractions of the gradient. The fraction carrying a single ribosome per mRNA molecule and co-sedimentating with the peak of empty ribosomes (80S peak on the profile shown in Figure 1) was omitted as it contained very low amounts of mRNAs (data not shown). Then, to accurately measure the size and compare the poly(A) tail lengths of polysomal fraction mRNAs, cDNAs were prepared using the Ligase-Mediated Poly(A) test (LM-PAT) described briefly in Figure 1 [20]. In the LM-PAT method, mRNA poly(A) tails are coated with an excess of oligo-(dT) primers in the presence of T4 DNA ligase. With this method, only poly(A)-tailed mRNAs are reverse transcribed, and thus, only a limited number of PCR cycles is required. In addition, it has been recently shown that the LM-PAT method accurately reflects the length of *in vitro* synthesized poly(A) tails [21]. We favored this poly(A)-directed approach upon other methods based on 3′ terminus extension of all RNA molecules, because ribosomal RNA constituted a major fraction in our RNA samples.

After amplification of the cDNA with gene specific forward primers, the distribution of poly(A) tail lengths in the polysomal fractions was analyzed by agarose gel electrophoresis. We analyzed MYC mRNA and a set of mRNAs including those encoding β-actin (ACTB), eukaryotic elongation factor 1G (EEF1G), ribosomal protein S6 (RPS6), cyclin D1 (CCND1) and c-Jun (JUN). The choice of this set of genes was dictated by some technical constraints such as the abundance of the mRNAs in the cultured HCT116 cells [22] and the absence of multiple polyadenylation sites that gave complex poly(A) tail profiles. For JUN mRNA, which harbors several polyadenylation sites, we chose the forward primer so that to amplify the poly(A) tail of the longest mRNA isoform which is also the most abundant isoform. In addition, the CCND1 and JUN mRNAs were also chosen because, like MYC mRNA, their 3′UTR contains AU-rich elements interacting with sequence-specific RNA-binding proteins [23, 24].

**Figure 2:**
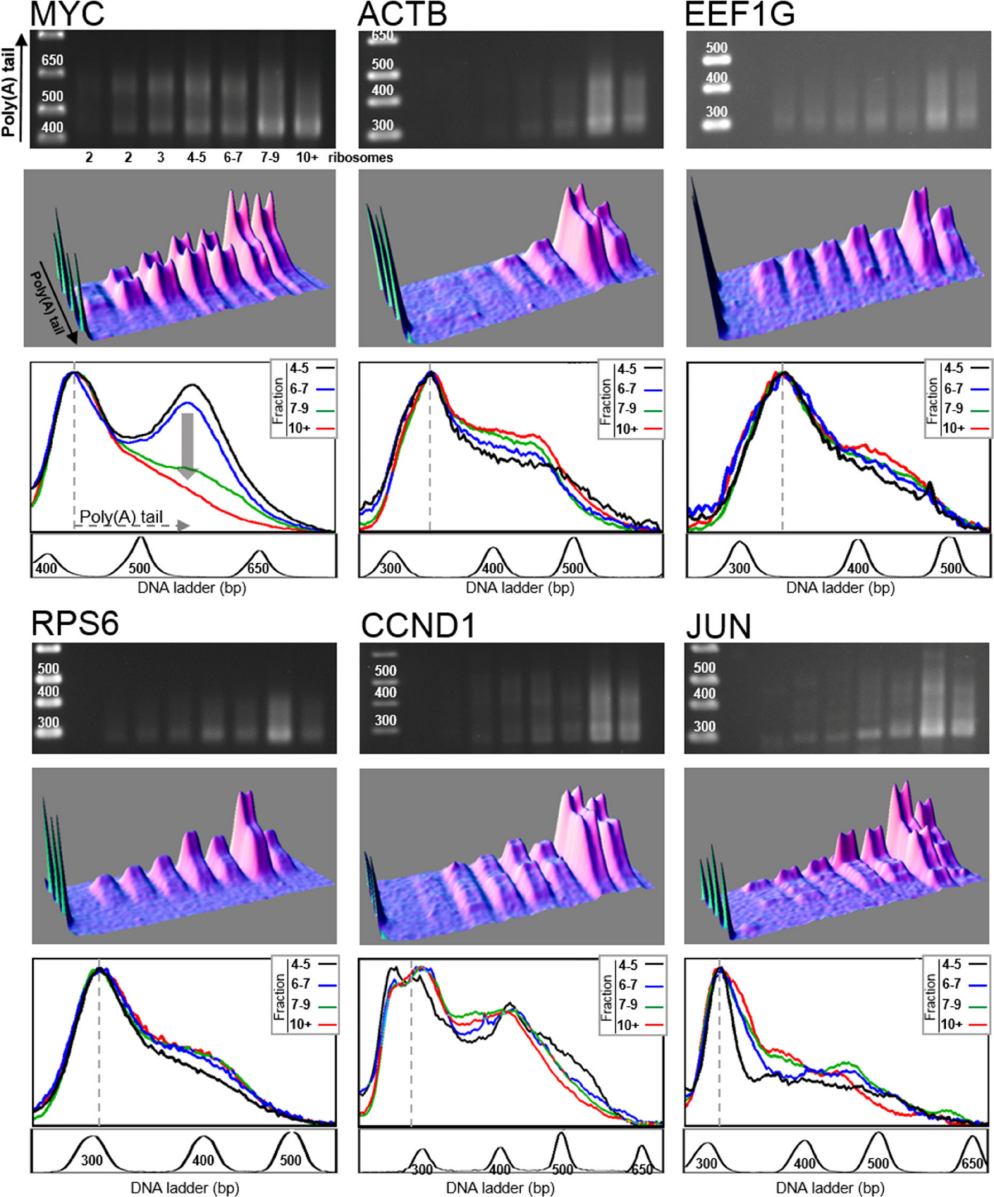
Distribution of poly(A) tail lengths of MYC, ACTB, EEF1G, RPS6, CCND1 and JUN mRNAs RNAs extracted from each fraction of a polysome fractionation experiment of HCT116 cell extract were subjected to LM-PAT analysis and cDNAs were amplified with gene specific forward primers. For the six genes presented, cDNAs were amplified from the same polysome fractionation experiment and LM-PAT reaction; only gene specific forward primers differ according to the analyzed gene. For each gene, MYC, ACTB, EEF1G, RPS6, CCND1 and JUN, the agarose gel with DNA ladder on the left (top panel), the 3D surface plot analysis of the agarose gel (middle panel) and the stacked image of the density profiles of the last four lanes corresponding to heavy polysomes (bottom panel) are shown. In the density profile panel, the fraction containing 4–5 ribosomes is in black, the fraction 6–7 ribosomes in blue, the fraction 7–9 ribosomes in green and the fraction with more than 10 ribosomes (10+) in red. For MYC gene, the vertical grey arrow on the density profile panel highlights the differences in poly(A) tail distribution between the four fractions. The density profiles of the gel lanes were performed without O. D. calibration. This allowed to normalize the profiles on the most intense band corresponding to mRNAs with minimal poly(A) tail (indicated by a dotted line). For each gene, a profile of the DNA ladder is presented below the density profile panel. Note that the experiment presented in this figure correspond to the control experiment of Figure 3 (cells electroporated with sh-Ctrl), see also Exp. 1 of Figure 4.

As shown in Figure 2 and in Table 1, analysis of the poly(A) tails revealed that the size of longest poly(A) tail varied among the investigated mRNAs (MYC, ACTB, EEF1G, RPS6, CCND1 and JUN), and ranged between 150 and 300 A residues, the longest being for JUN mRNA and the shortest for RPS6 mRNA. Whereas, for some genes, poly(A) tails appeared as a continuous smear on agarose gel, whatever the polysomal fraction considered, for some others, a distinct band corresponding to long poly(A) tails is visible. This is particularly clear with MYC for which two poly(A) species are well separated on the density profiles of fractions 4–5 and 6–7 (Figure 2, bottom panel for MYC transcript). To be sure that these two peaks correspond indeed to long and short poly(A) tail species and not to two mRNA isoforms, the LM-PAT assay products obtained from the fraction harboring two well-separated peaks, i. e., fraction 4–5 (Figure 2), were sequenced. We also sequenced the fraction 7–9 from ACTB LM-PAT experiment shown in Figure 2. The sequencing results unambiguously demonstrated that the two peaks observed for MYC mRNA or for ACTB mRNA were due to differences in the length of the poly(A) tail (Supplementary Figure 1). We can thus reasonably assume that for all the target genes, the DNA smears observed on the agarose gel are due to differences in poly(A) tail lengths.

**Table 1:** Lengths of the longest poly(A) tail

Gene name	Distance between gene specific primer 5′ end and mRNA poly(A) site^a^ (nt)	Approximate length of the longest poly(A) tail (nt)
MYC	420	230
ACTB	268	220
RPS6	234	150
EEF1G	264	170
JUN	272	300
CCND1	248	260

^a^The PCR fragment length for minimal poly(A) tail (Figure 2) is 30 nt longer due to the presence of the anchor primer.

We next compared the distribution of poly(A) tail lengths in the last four fractions, corresponding to heavy polysomes. The density profile analysis of the gel lanes (bottom panel for each gene in Figure 2) was performed without O. D. calibration. This allowed us to normalize the profiles to the most intense band which corresponds to the mRNAs with minimal poly(A) tail. Note that, in the experimental conditions we used, the intensity of the LM-PAT shortest cDNA amplification band is proportional to the amount of mRNA present in the analyzed fraction as revealed by the correlated intensity of the band obtained by direct RT-PCR on the extracted mRNA (Supplementary Figure 2). Interestingly, the density profile analysis shows that, with the exception of MYC mRNA, there is a quite good overlap between the profiles of the different fractions for all examined transcripts: ACTB, EEF1G, RPS6, CCND1 and JUN mRNAs (Figure 2, bottom panel for each gene). This overlap shows that the poly(A) tail length distribution is relatively constant among the different polysomal fractions, i.e., whatever the number of harbored ribosomes.

For MYC mRNA, the superimposition of the density profiles shows that the poly(A) tail length distribution clearly changed in the last two lanes corresponding to the fractions containing mRNAs translated by more than 7 ribosomes (Figure 2, bottom panel of MYC, changes are highlighted by a vertical grey arrow). In these two heaviest fractions, which contained the most actively translated mRNAs, the length of the poly(A) tails was decreased. Indeed, the peak corresponding to the longest poly(A) tail, which is clearly visible in fractions 4–5 and 6–7, has disappeared in fractions 7–9 and 10+. The accumulation of MYC mRNAs with short poly(A) tail in these last two fractions suggested that some MYC mRNA molecules were deadenylated concomitantly to their translation. Therefore, these results suggested that the other tested mRNA species did not undergo the same deadenylation process.

The heaviest fractions, i.e., with the highest number of ribosomes on the mRNA, are also those with the highest probability that some ribosomes have completed the open reading frame translation and are therefore those containing the highest proportion of mRNAs with ribosomes undergoing translation termination. Thus, we wondered whether MYC mRNA was co-translationally deadenylated once it has experienced translation termination.

### Influence of translation termination on MYC mRNA deadenylation

**Figure 3:**
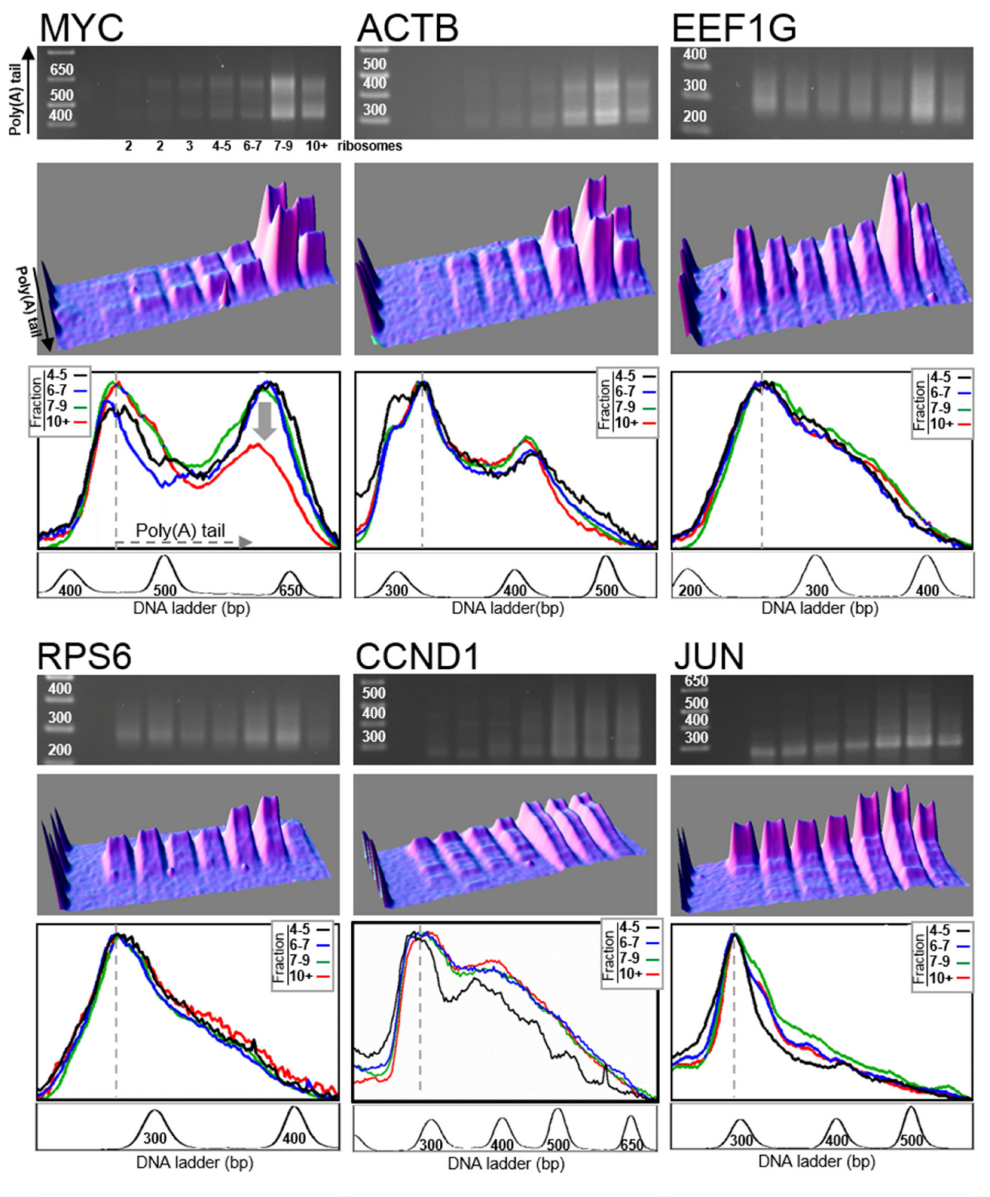
Distribution of poly(A) tail lengths in eRF3a-depleted cells Human HCT116 cells were electroporated with a plasmid expressing small interfering RNAs targeting eRF3a mRNA (sh-3a1). Three days after electroporation, the cell extract was fractionated on sucrose gradient and RNAs were extracted from the polysomal fractions. RNAs were subjected to LM-PAT analysis and cDNAs were amplified with gene specific forward primers. For the six genes presented, cDNAs were amplified from the same polysome fractionation experiment and LM-PAT reaction; only gene specific forward primers differ according to the analyzed gene. For each gene, MYC, ACTB, EEF1G, RPS6, CCND1 and JUN, the agarose gel with DNA ladder on the left (top panel), the 3D surface plot analysis of the agarose gel (middle panel), and the stacked image of the density profiles of the last four lanes corresponding to heavy polysomes (bottom panel) are shown. In the density profile panel, the fraction containing 4–5 ribosomes is in black, the fraction 6–7 ribosomes in blue, the fraction 7–9 ribosomes in green and the fraction with more than 10 ribosomes (10+) in red. The density profiles of the gel lanes were performed without O. D. calibration allowing to normalize the profiles on the most intense band corresponding to mRNAs with minimal poly(A) tail (indicated by a dotted line). For each gene, a profile of the DNA ladder is presented below the density profile panel.

In order to evaluate the influence of the translation termination process on MYC mRNA deadenylation, we performed the same poly(A) tail analysis on mRNAs extracted from cells depleted in translation termination factor eRF3a, which is the active isoform of the translation termination factor eRF3 in mammalian cells [25]. We have previously shown that, in mammalian cells, the depletion of eRF3a caused a concomitant reduction of eRF1 level by decreasing its stability [25]. Thus, depletion of eRF3a induced a defect in the whole translation termination complex. Human HCT116 cells were electroporated with plasmids expressing the interfering RNAs sh-3a1 or sh-3a9 both targeting eRF3a mRNA. Three days after electroporation, cell lysates were subjected to polysomal fractionation and LM-PAT analysis was performed on RNA extracted from each polysomal fraction. eRF3a depletion was ascertained by Western blot analysis and RT-PCR (see Supplementary Figure 3). Representative experiments of poly(A) tail length distribution analysis of eRF3a-depleted samples are presented in Figure 3 for sh-3a1 and in Supplementary Figure 4 for sh-3a9 electroporated cells. The set of genes studied in eRF3a-depleted cells was the same as that mentioned above for non-depleted cells. Similarly to the non-depleted cells, there is a quite good overlap of the density profiles of the last four fractions, including the heaviest fractions, for ACTB, EEF1G, RPS6, CCND1 and JUN mRNAs (Figure 3, bottom panels). This shows that the translation termination defect induced by eRF3a depletion did not modify the poly(A) tail length distribution for these mRNAs.

**Figure 4:**
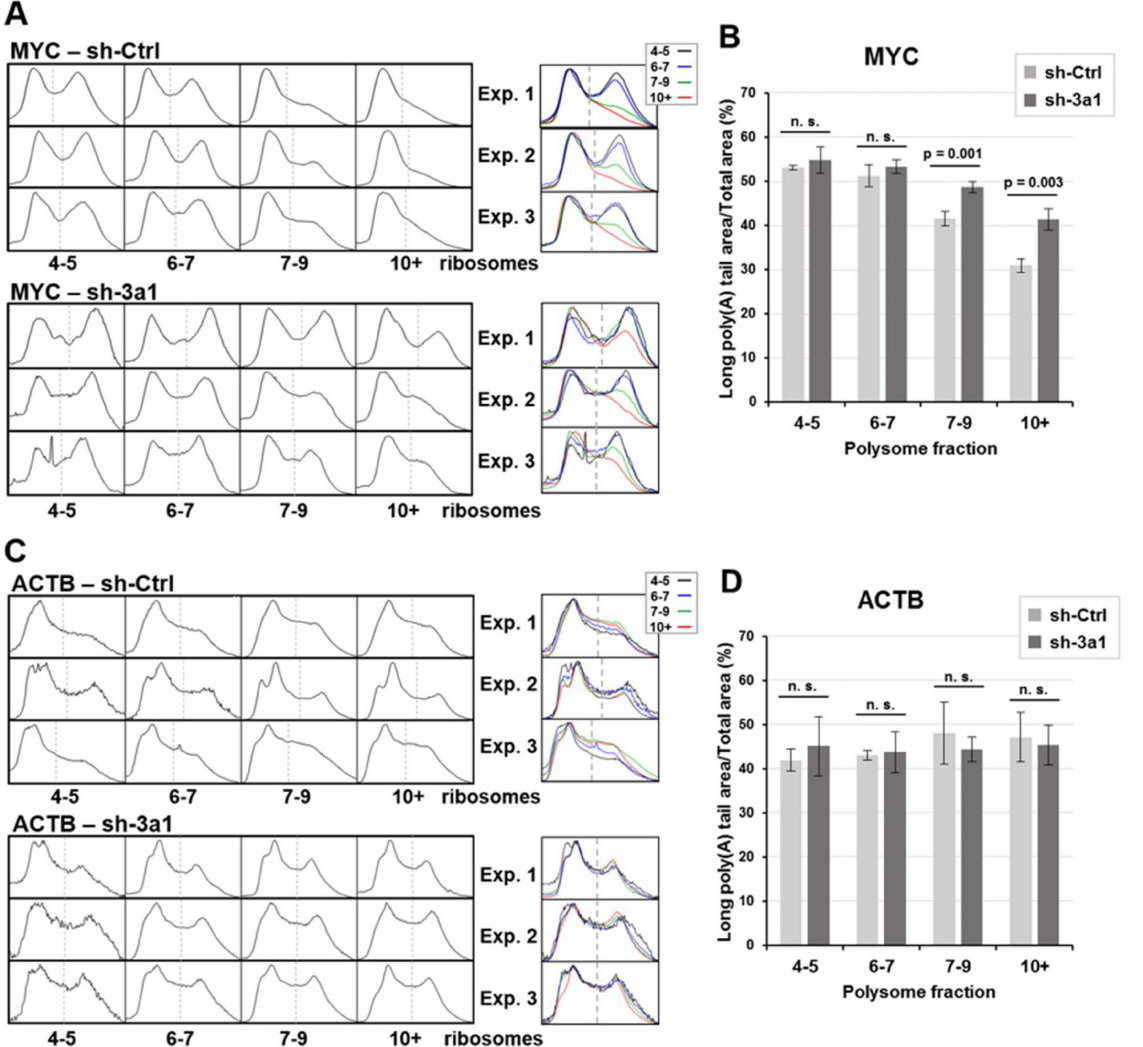
Density profiles of MYC and ACTB poly(A) tails and poly(A) peak area measurements for three independent experiments in eRF3a-depleted and control cells The montage and superimposition of the density profiles of the last four fractions (4–5, 6–7, 7–9 and 10+ ribosomes) of 3 polysome fractionation experiments with either eRF3a-depleted (sh-3a1) or control (sh-Ctrl) cell extracts are shown for MYC mRNA (**A**) and ACTB mRNA (**C**). Agarose gel and 3D surface plot images of experiment 1 (Exp.1) are shown in Figure 2 and 3 for control and eRF3a-depleted cells, respectively. Agarose gel and 3D surface plot images of experiments 2 (Exp. 2) and 3 (Exp. 3) are shown in Supplementary Figure 6. For MYC – sh-3a, exp. 3, the narrow peak in the middle of fraction 4-5 profile is due to a dust dot in the agarose gel (see the corresponding agarose gel in Supplementary Figure 6). On each density profile, the dash line indicates the boundary of the long poly(A) peak area used for the peak area measurements. For each experiment, the dash line position was determined on the fraction presenting a clear separation of the two peaks (usually fraction 4–5) and set at the same position for the other fractions. The poly(A) peak areas of the density profiles were measured using ImageJ software and the ratio of long poly(A) tail area on total profile area was calculated for the four fractions (4–5, 6–7, 7–9 and 10+ ribosomes) of the 3 polysome fractionation experiments with either control (sh-Ctrl) cells or eRF3a-depleted cells (sh-3a1). For MYC mRNA (**B**) and ACTB mRNA (**D**), bars represent the mean values of the three experiments. Error bars indicate the standard deviation. Statistical significance of differences between sh-Ctrl and sh-3a1 were calculated with paired Student's *t*-test. For statistically significant differences, *p*-values are indicated above the bars; *p*-values ≥ 0.05 were considered as not statistically significant (n. s.).

In the case of MYC mRNA, the decrease in poly(A) tail length observed in the heaviest fractions containing mRNAs translated by more than 7 ribosomes was clearly attenuated in eRF3a-depleted cells (compare the superimposition of the density profiles of fractions 7–9 and 10+ between Figures 2 and 3, bottom panels). This result suggested that MYC mRNA deadenylation was substantially reduced in eRF3a-depleted cells. Similar changes in poly(A) tail length distribution were repeatedly obtained in HCT 116 cells as shown either for three independent electroporation experiments using sh-3a1 (Figure 4), or when another sh-RNA directed against eRF3a was used (sh-3a9, Supplementary Figure 4). Moreover, similar results were also obtained when the human HEK293 cell line served as the host cells (Supplementary Figure 5).

To ascertain the differences in MYC poly(A) tail length between control and eRF3a-depleted cells, we measured the poly(A) tail areas on the density profiles of MYC polysomal fractions from all three independent electroporation experiments (Figure 4). As a comparison, the same measurements were performed for ACTB mRNA fractions. Only the density profiles of the last four polysomal fractions corresponding to heavy polysomes are shown (Figure 4A, for MYC and Figure 4C, for ACTB). First, in all the fractions, we determined the boundary of the long poly(A) tail area (indicated by a thin dashed line in Figure 4A and 4C) on the density profiles from the three experiments. Thus, the fraction (usually fraction 4–5) showing a clear separation of the poly (A) tail length into two peaks (corresponding to short and long poly(A) tails), was used to determine the position of the boundary between both species. This reference position was used to set this boundary at the same position for the other fractions. Then, for each density profile, the poly(A) areas were measured and the ratio of long poly(A) peak area on the total profile area was calculated for the four fractions (Figures 4B for MYC and 4D for ACTB). The comparison of this ratio between the two experimental conditions confirms that, in the largest polysomes (fractions 7–9 and 10+), the poly(A) tail of MYC mRNA is significantly longer in eRF3a-depleted cells than in control cells (for fraction 10+, there is a 33.6 % increase of long poly(A) tail area for eRF3a-depleted cells compared to control cells, with *p*-value = 0.0034 for three independent experiments, Figure 4B). Conversely, poly(A) peak area measurement of ACTB mRNA showed that poly(A) tail length distribution is similar for all fractions and is not affected by a translation termination defect (Figure 4D).

Therefore, we conclude that shortening of the MYC mRNA poly(A) tail is slowed when translation termination is altered, and that the co-translational deadenylation of MYC mRNA requires translation termination to proceed.

## DISCUSSION

For most of mRNA decay pathways, the earliest step in cytoplasmic mRNA degradation is deadenylation, a progressive shortening of the mRNA poly(A) tail by deadenylases [9, 26]. However, for the vast majority of mRNA species very little is presently known about the specific mechanism of their deadenylation and when this process takes place: either independently of translation, following disassembly of translating polysomes, or during the active translation process. In this study, we attempted to answer these questions by analyzing the poly(A) tail length distribution of various mRNAs isolated from the polysomal fractions of polysome profiling experiments. For all mRNAs studied, poly(A) tails are highly heterogeneous in length as previously reported [17, 18]. While indeed, for some genes, the poly(A) tails appeared as a continuous smear on agarose gel, whatever the polysomal fraction considered, for some others, particularly MYC and ACTB, a band corresponding to long poly(A) tails is clearly visible.

The global investigations allowing to measure poly(A) tail sizes at the genomic scale have shown that poly(A) tail length and translation efficiency are decoupled in non-embryonic cells [17, 18]. Considering that translation efficiency is proportional to the number of ribosomes per transcript, our analysis shows that, for all of the genes tested with the only exception of MYC, the poly(A) tail length does not vary with the number of ribosomes carried by the mRNA, which is in good agreement with the recent findings mentioned above.

In the case of MYC mRNA, the poly(A) tail length distribution clearly changed along the polysome profile. Up to the fraction containing mRNAs carrying 6–7 ribosomes, the long poly(A) tails are relatively homogeneous in length, clustered in a peak at ~230 A residues. This peak progressively disappears in the last two fractions containing mRNAs translated by more than 7 ribosomes (Figure 2). This result suggests that for MYC mRNA the length of the poly(A) tail is correlated with translation efficiency and that the most actively translated mRNAs are subjected to poly(A) tail shortening by deadenylation. Our experiments confirm the result originally presented by Laird-Offringa *et al.* linking shortening of MYC mRNA poly(A) tail and ongoing translation [19]. Our study goes further and allows to precisely associate MYC mRNA deadenylation with polysomes enriched in mRNAs completing translation. Indeed, the only substantial difference between mRNA molecules translated by a small number of ribosomes and those translated by more than 7 ribosomes is that, in the latter case, a higher number of ribosomes completed the open reading frame translation and reached the stop codon. This raises the possibility that deadenylation of MYC mRNA is coupled with the last step of translation, namely, translation termination. Our results showing that a translation termination defect reduces the shortening of MYC mRNA poly(A) tail support this hypothesis (Figures 3 and 4). Besides, it is noticeable that eRF3a depletion had no effect on the poly(A) tail length distribution of the other genes.

What could be the mechanism coupling translation termination and MYC mRNA deadenylation? It has been proposed that, upon translational termination, recruitment of translation termination complex to the ribosomes permits the binding of the deadenylase complexes to the 3′ end of the poly(A) tail, thus coupling mRNA deadenylation with translation termination [27]. However, this hypothesis concerns the general mechanism of deadenylation involving the binding of eRF3 on PABP and the recruitment of deadenylation complexes via their competitive binding to PABP. If this hypothesis applies to our experiments, we should have likely observed poly(A) tail shortening for other mRNAs besides MYC, which is not the case. Recently, mammalian MYC mRNA has been shown to escape the two-step general deadenylation pathway and to be degraded through a specific decay process involving the TOB-CAF1 deadenylation complex interacting with the sequence-specific RNA-binding proteins CPEB [13]. The binding of CPEB to CPEs (Cytoplasmic Polyadenylation Element) present in MYC mRNA 3′UTR allows the recruitment of TOB-CAF1 complex. Our hypothesis to explain the link between MYC mRNA deadenylation and translation termination would be that translation termination facilitates the recruitment of deadenylase complex on CPEB. Nevertheless, the case of MYC mRNA seems to be quite particular as accelerated deadenylation involving CPEB was not described for other CPE-containing mRNAs. For instance, reporter mRNAs appended with the 3′UTR of another CPEB-target, such as JUN 3′UTR, were not affected by overexpression of CPEB [13]. This is quite coherent with our experiments in which JUN mRNA poly(A) tail length distribution is not changed along the polysome gradient profile and is also not sensitive to eRF3a depletion (Figures 2 and 3). In addition, our results with CCND1 mRNA which also harbors AREs in its 3′UTR [23], suggest that the presence of AREs is not the determinant element of the particular poly(A) tail length distribution of translated MYC mRNA either.

In conclusion, we show that co-translational deadenylation of mRNAs is not a prevalent process but likely concerns some genes that use uncommon deadenylation pathways.

## MATERIALS AND METHODS

### Cell culture and cell electroporation

The HCT116 cell line (ATCC CCL-247) was maintained in McCoy medium (Invitrogen) supplemented with 10% fetal calf serum, 1 mM sodium pyruvate, 100 μg/ml streptomycin and 100 units/ml penicillin at 37°C under 5% CO2 atmosphere. HEK293 cells (ATCC CRL-1573) were cultured in Dulbecco modified Eagle's medium (Invitrogen) supplemented with 10% fetal calf serum, 100 μg/ml streptomycin, and 100 units/ml penicillin. eRF3a silencing by plasmid expressing small interfering RNAs targeting eRF3a mRNA (sh-3a1 and sh-3a9) was previously described [28]. Plasmid pSUPERcontrol with the non-silencing shRNA sequence (5′-ATTCTCCGAACGTGTCACG-3′) was used as negative control (sh-Ctrl). Electroporation of cells was performed with a Gene pulser II electroporation system (Bio-Rad) using 4.8 × 10^6^ cells and 20 μg of plasmid DNA. Electroporated cells were collected 72 h after electroporation.

### Polysome profiling and RNA isolation

Polysome profiling experiments were performed according to Verrier and Jean-Jean [29] with minor modifications. Three days after electroporation, two 100-mm plates of either HCT116 or HEK293 cells at 70% cell confluence were used for polysome fractionation. Cell extracts were loaded onto 15–50% sucrose gradients and subjected to ultracentrifugation. After centrifugation, optical density (O. D.) at 254 nm was monitored by pumping the gradient through a Retriever 500 (Teledyne Isco) fraction collector. The flow rate was optimized in order to collect ~15 fractions of 0.8 mL each. Afterward, each fraction was precipitated with 1.2 mL of isopropanol and kept at −80°C for further RNA analysis. For RNA isolation, the polysomal fractions of the sucrose gradients (Figure 1) were subjected to centrifugation at 18,000 × g for 1 hr, then the pellets were rinsed with 1 mL of 80% ethanol, dried and resuspended in 350 μl of RAI buffer of NucleoSpin RNA II kit (Macherey-Nagel) containing 1% ß-mercaptoethanol (v/v). RNA was purified according to the manufacturer's instructions and eluted in 40 μl of RNase-free water.

### LM-PAT cDNA synthesis

LM-PAT experiments were performed according to Sallés *et al.* [20]. Firstly, 2 μl of 5′-phosphorylated poly(dT)12-18 oligonucleotide (10 ng/μl in RNase-free water) was added to 5 μl of each of the purified RNA fractions in order to saturate the entire poly(A) tails. The mixture was heat-denatured at 65°C for 10 min and immediately transferred to a 42°C water bath. Then, 13 μl of a pre-warmed (42°C) mixture containing 2 μl of 10× RT buffer from the SuperScript First-Strand Synthesis System for RT-PCR (Life Technologies), 1 μl of dNTP mix (10 mM each), 4 μl of 25 mM MgCl_2_, 2 μl of 0.1 mM DTT, 1 μl of RNase OUT, 1 μl of 10 mM ATP, and 1.7 μl of T4 DNA ligase (New England Biochemicals) corresponding to 10 Weiss units was added with gentle mixing. After incubation for 30 min at 42°C and while at 42°C, 1 μl of an excess of oligo(dT) anchor oligonucleotide (5′-GCGAGCTCCGCGGCCGCG-T12-3′) (200 ng/μl) was added and the reactions were further incubated at 12°C for 2 h. The oligo(dT) anchor mainly ligates specifically to the 5′-phosphorylated oligo(dT) hybridized at the 3′ most end of the poly(A) tail. The reactions were then transferred to 42°C and pre-incubated for 2 min, before adding 1 μl (200 units) of SuperScript II reverse transcriptase (Life Technologies). The reverse transcription reaction was then performed by incubation at 42°C for 1 h. Finally, the reverse transcriptase was heat inactivated at 70°C for 15 min.

The short incubation at 42°C before the addition of the reverse transcriptase minimizes priming of unligated oligo (dT) anchor at the very 5′-end of the poly(A) tail. Nevertheless, the small amount of oligo (dT) anchor priming at the 5′-end of the poly(A) tail allows to measure the length of the cDNA fragment with minimal poly(A) tail and hence to estimate the length of the poly(A) tail [20].

### PAT-PCR analysis

PCR forward primers for specific genes (Table 2) were chosen according to the recommendations of Sallés and Strickland [30]. They were designed close to the 3′ end of the mRNA in order to obtain 250 to 650 base pair long DNA fragments after amplification. Standard PCR was performed with GoTaq^®^ G2 Green Master Mix (Promega), with 0.5 μM forward gene specific primer, 0.5 μM oligo(dT) anchor reverse primer and 1–2 μl of template PAT cDNA in a 25 μl reaction volume. Typically, the PCR conditions were 3 min at 95°C (initial denaturation), followed by ~30 cycles of 30 s at 95°C, 45 s at 60°C, 1 min at 72°C, ending with a 7-min final extension at 72°C. The cycle number was dependent on the abundance of the transcript of interest in the sample and ranged between 25 and 33 cycles (25 cycles for ACTB, EEF1G and RPS6; 30 cycles for MYC and CCND1; 33 cycles for JUN). PCR samples were then loaded onto a 2% agarose gel in Tris-Borate EDTA buffer and electrophoresis was performed for 3 h at 5 V/cm. After ethidium bromide staining and extensive washing of the gel, photographs were taken using a UV transilluminator Gel Doc XRT (BioRad). The image of the gel was further processed with ImageJ software. Surface plots were obtained using the Interactive 3D Surface Plot option of the ImageJ 1.49v program. Gel lane density profiles were obtained using the Gel Analyzer menu of ImageJ and identical rectangle areas were selected to analyze each gel lane. We also used the uncalibrated O. D. option in order to compare the relative intensities of poly(A) tail lengths. Images of lane profiles were then stacked to superimpose the profiles.

**Table 2:** Gene specific forward primers used for LM-PAT experiments

Gene name	Accession Number	Primer (5′ to 3′)
MYC	NM_002467.4	AACCTCACAACCTTGGCTGAGTCTTG
ACTB	NM_001101.3	ACAGGGGAGGTGATAGCATTGCTT
RPS6	NM_001010.2	CGTATTGCTCTGAAGAAGCAGCGTA
EEF1G	NM_001404.4	TTGCCTTTCCGCTGAGTCCAGATTG
JUN	NM_002228.3	GGACAGCCCACTGAGAAGTCAAACA
CCND1	NM_053056.2	GTCCTGGATGTTGTGTGTATCGAGAG

## SUPPLEMENTARY MATERIALS FIGURES


