# Supplementary material for: Translation termination-dependent deadenylation of MYC mRNA in human cells
Source: Oncotarget. 2018 May 25;9(40):26171–82. doi: 10.18632/oncotarget.25459 (PMC5995228; doi:10.18632/oncotarget.25459)
Supplement: Supplementary file 1 [file oncotarget-09-26171-s001.pdf]

Translation termination-dependent deadenylation of MYC mRNA in human cells

SUPPLEMENTARY MATERIALS

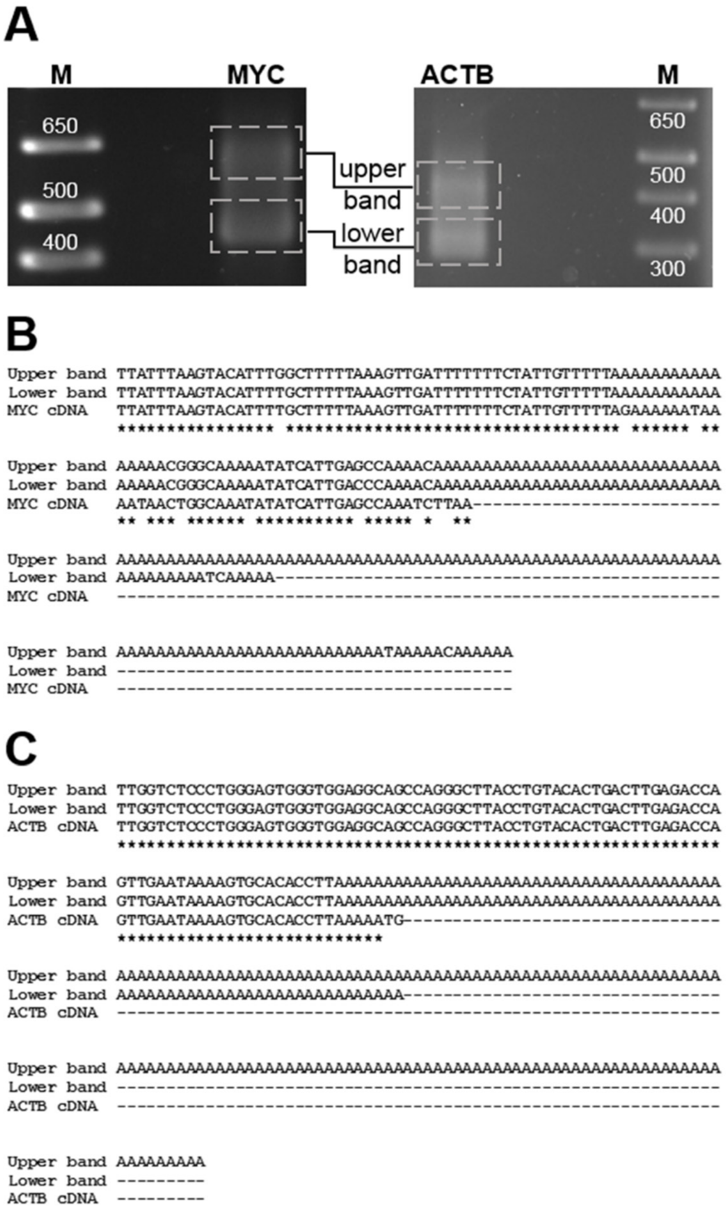

Supplementary Figure 1: Sequencing of MYC and ACTB poly(A) tails. (**A**) LM-PAT reactions of fractions 4-5 for MYC and 7-9 for ACTB shown in Figure 2 were submitted to electrophoresis on a 2% agarose gel. M: DNA ladder (bp). For each fraction, two bands (upper and lower bands) were excised from the gel and submitted to electroelution. After ethanol precipitation, the recovered DNA was directly sequenced. Sequencing results for MYC (**B**) and ACTB (**C**). The sequences of upper and lower bands were aligned to that of wild type mRNA (Accession numbers: MYC NM_002467.4 and ACTB NM_001101.3) using ClustalW.

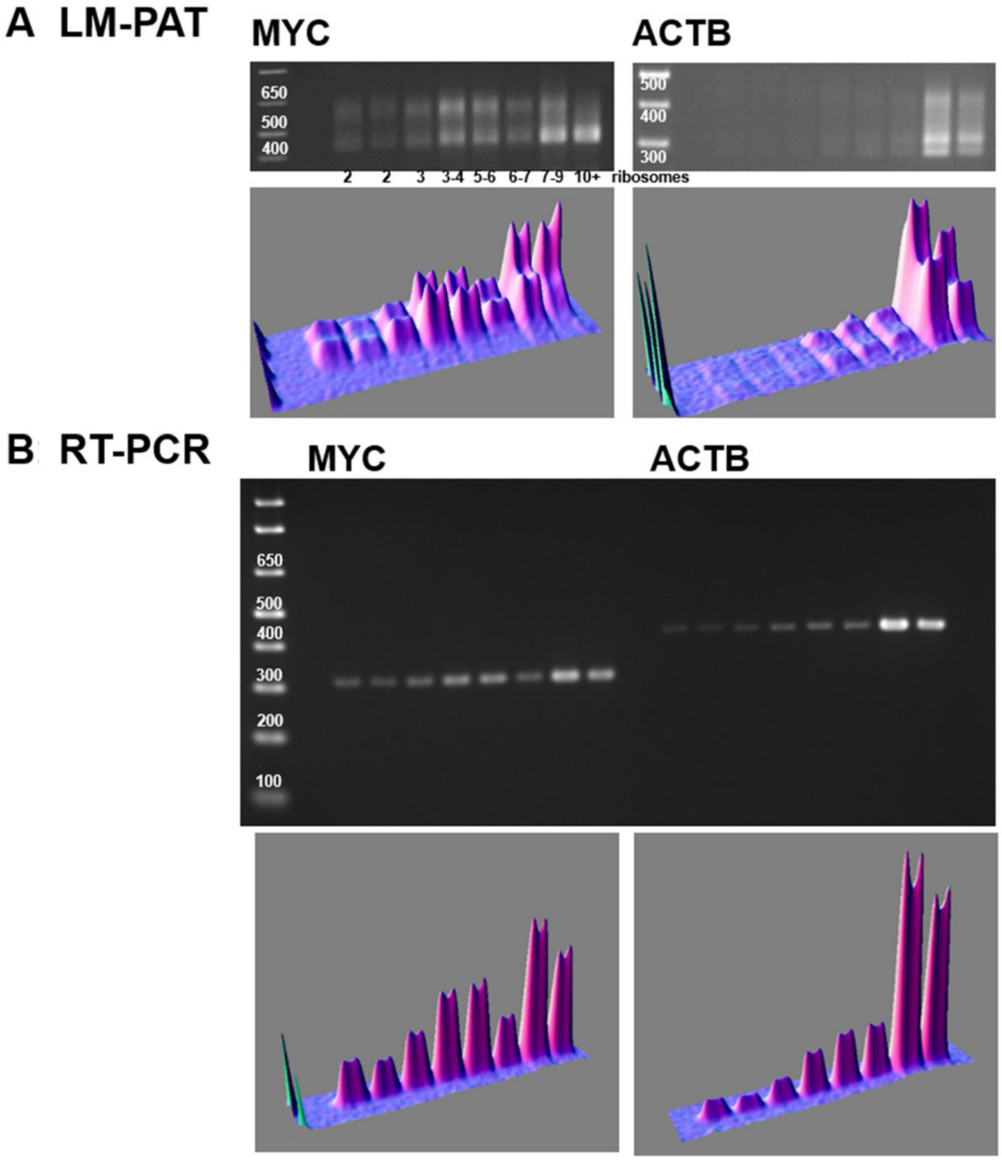

Supplementary Figure 2: Agarose gel and 3D surface plot images for MYC and ACTB mRNAs. RNAs extracted from the polysomal fractions of polysome profiles were subjected either to LM-PAT (**A**) or to RT-PCR (**B**). LM-PAT was performed as described in Materials and Methods section. For RT-PCR, the reverse transcription reaction was performed with the SuperScript First-Strand Synthesis System for RT-PCR (Life Technologies) as recommended in the manufacturer's instructions, using oligo(dT) and 5 µl of each of the purified RNA fractions and PCR was performed with primers 5′AACCGAAAATGCACCAGCCCCAG3′ and 5′ TCCTTACGCACAAGAGTTCC-GTAGC3′ for MYC and 5′AGAAGAGCTACGAGCTGC-CTGAC3′ and 5′CGCCTAGAAGCA-TTTGCGGTGGA3′ for ACTB. These couples of primers were chosen in order to amplify a fragment of the coding sequence for both genes: a 309-bp fragment for MYC and a 416-bp fragment for ACTB. PCR conditions were 3 min at 95° C (initial denaturation), followed by 21 cycles (ACTB) or 24 cycles (MYC) of 30 s at 95° C, 30 s at 60° C, 1 min at 72° C, ending with a 7-min final extension at 72° C. Surface plot images highlight the proportionality of the LM-PAT signal with the amount of mRNA present in the analyzed fraction.

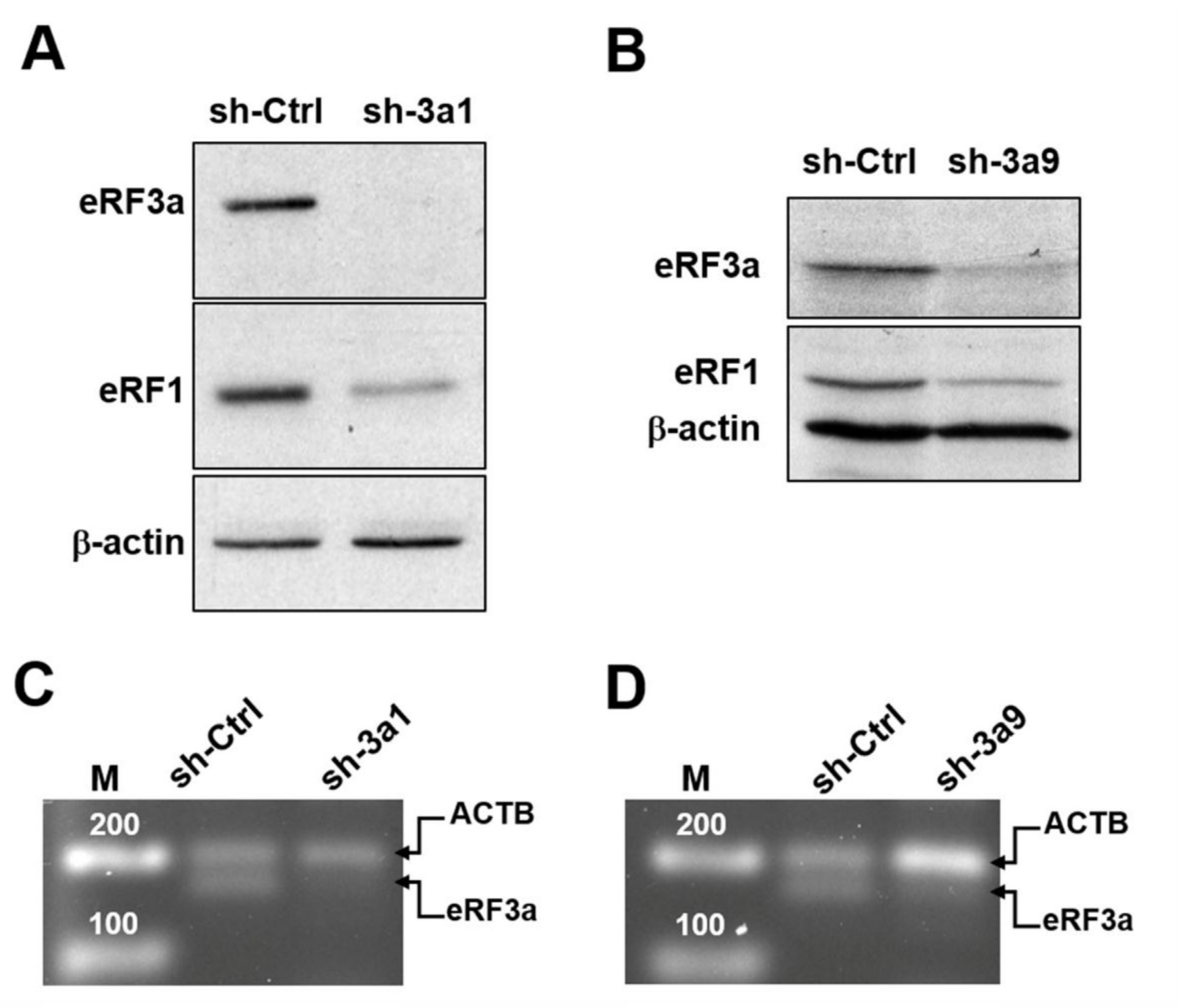

Supplementary Figure 3: Western blot and RT-PCR analyses of eRF3a depletion in HCT116 cells. Cells were electroporated either with control plasmid (sh-Ctrl) or with plasmids expressing sh-RNA directed against eRF3a mRNA, sh-3a1 in (**A** and **C**) and sh-3a9 in (**B** and **D**). For Western blotting (A and B), cells were lysed by sonication on ice, centrifuged for 20 min at 16,000 × g and supernatant retained as cell extracts. For each sample, 30 µg of total protein were loaded on polyacrylamide gel and subjected to electrophoresis. Western blotting was then performed as described in reference 26 (main text). β-actin served as loading control. As shown in panels A and B, eRF3a depletion induces a concomitant eRF1 depletion (see ref. 26). For RT-PCR analysis (C and D), RNA was extracted from electroporated cells and the reverse transcription reaction was performed with the SuperScript First-Strand Synthesis System (Life Technologies) as recommended in the manufacturer's instructions, using oligo(dT) and 1 µg of each of the purified RNA fractions. Multiplexed PCR reactions were performed with primers 5′GAGGAGGAAGAGGAAATCCC3′ and 5′TCCTTTTGTCAACCAT-TCCA3′ for eRF3a (0.5 µM final concentration for each primer) and 5′TCCCTGGAGAAGAG-CTACGA3′ and 5′AGCACTGTGTTGGCGTACAG3′ for ACTB (0.05 µM final concentration for each primer). PCR conditions were 3 min at 95° C, followed by 30 cycles of 30 s at 95° C, 30 s at 51° C, 1 min at 72° C, ending with a 7-min final extension at 72° C. DNA fragments were then subjected to 2% agarose gel electrophoresis. M: DNA ladder (bp).

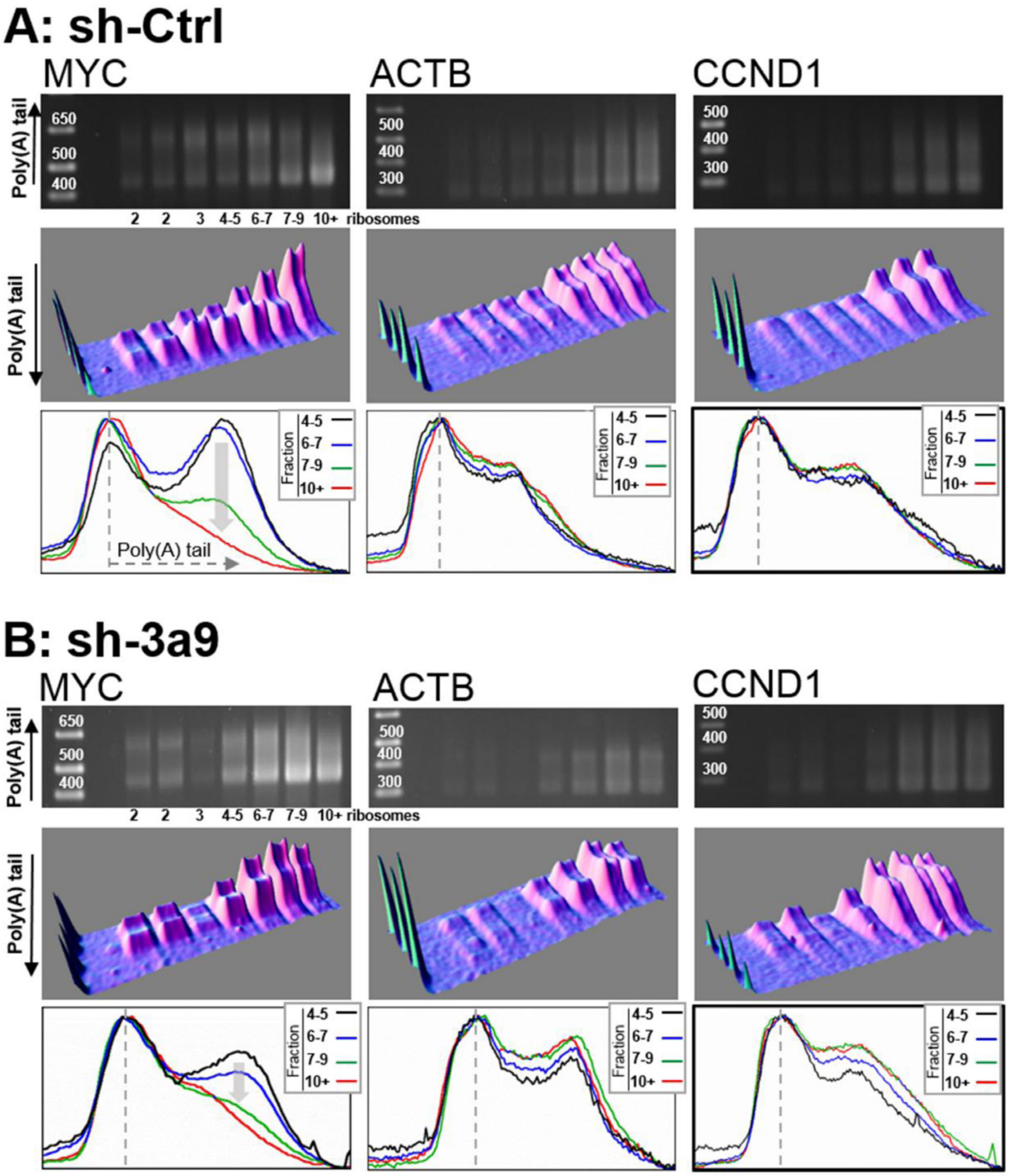

Supplementary Figure 4: Agarose gel, 3D surface plot and density profiles for MYC, ACTB and CCND1 mRNAs. HCT116 cells were electroporated either with control plasmid (**A**: sh-Ctrl) or plasmid expressing a sh-RNA directed against eRF3a mRNA (**B**: sh-3a9). For MYC mRNA, the vertical grey arrow on the density profiles highlights the modification of poly(A) tail length distribution between fractions 4-5, 6-7, 7-9 and 10+.

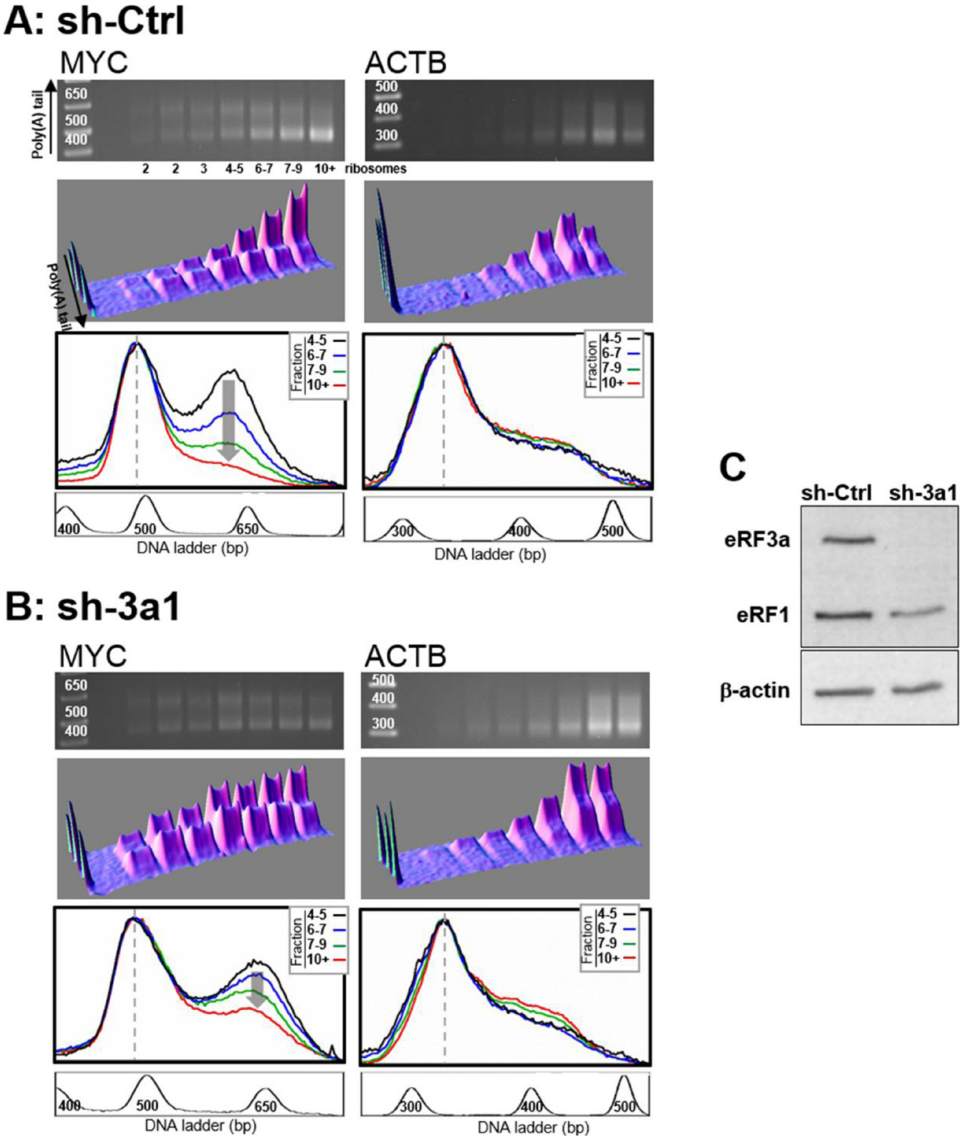

Supplementary Figure 5: Polysome fractionation and LM-PAT experiments in HEK293 cells. HEK293 cells were electroporated either with control plasmid (**A**: sh-Ctrl) or plasmids expressing sh-RNA directed against eRF3a mRNA (**B**: sh-3a1). Agarose gel, 3D surface plot and density profiles for MYC and ACTB mRNAs are shown. For MYC mRNA, the vertical grey arrow on the density profiles highlights the modification of poly(A) tail length distribution between fractions 4-5, 6-7, 7-9 and 10+. For each gene, a profile of the DNA ladder is presented below the density profile panel. Western blot analysis (**C**) of HEK293 cell extracts electroporated with sh-Ctrl or sh-3a1 using antibodies directed against eRF3a, eRF1 and β-actin (the membrane was co-incubated with anti-eRF3a and anti-eRF1 antibodies).

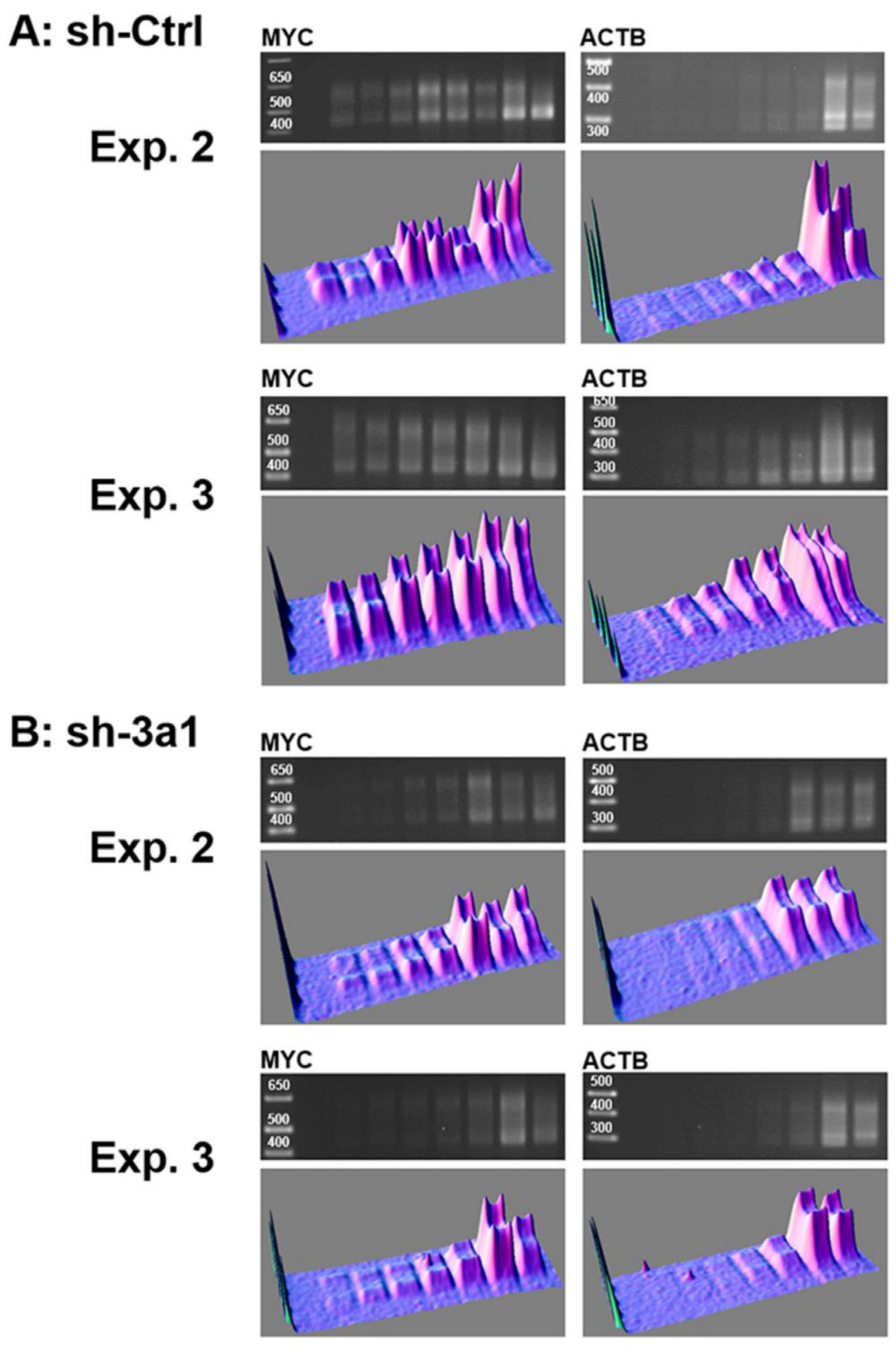

Supplementary Figure 6: Agarose gel and 3D surface plot images for MYC and ACTB mRNAs, corresponding to the density profiles of experiments 2 and 3 (Exp. 2 and Exp. 3, respectively) shown in Figure 4. Cells were electroporated either with control plasmid (**A**: sh-Ctrl) or plasmid expressing sh-RNA directed against eRF3a mRNA (**B**: sh-3a1).
